# Intravenous Bone Marrow Mononuclear Cells Transplantation Improves the Effect of Training in Chronic Stroke Mice

**DOI:** 10.3389/fmed.2020.535902

**Published:** 2020-11-26

**Authors:** Yuko Ogawa, Yuka Okinaka, Yukiko Takeuchi, Orie Saino, Akie Kikuchi-Taura, Akihiko Taguchi

**Affiliations:** Department of Regenerative Medicine Research, Foundation for Biomedical Research and Innovation at Kobe, Hyogo, Japan

**Keywords:** stroke, cerebral infarction, bone marrow mononuclear cell, cell therapy, chronic

## Abstract

There is no effective treatment for chronic stroke if the acute or subacute phase is missed. Rehabilitation alone cannot easily achieve a dramatic recovery in function. In contrast to significant therapeutic effects of bone marrow mononuclear cells (BM-MNC) transplantation for acute stroke, mild and non-significant effects have been shown for chronic stroke. In this study, we have evaluated the effect of a combination of BM-MNC transplantation and neurological function training in chronic stroke. The effect of BM-MNC on neurological functional was tested four weeks after permanent middle cerebral artery occlusion (MCAO) insult in mice. BM-MNC (1 × 10^5^cells in 100 μl PBS) were injected into the vein of MCAO model mice, followed by behavioral tests as functional evaluations. Interestingly, there was a significant therapeutic effect of BM-MNC only when repeated training was performed. This suggested that cell therapy alone was not sufficient for chronic stroke treatment; however, training with cell therapy was effective. The combination of these differently targeted therapies provided a significant benefit in the chronic stroke mouse model. Therefore, targeted cell therapy *via* BM-MNC transplantation with appropriate training presents a promising novel therapeutic option for patients in the chronic stroke period.

## Introduction

Stroke is a medical condition in which poor blood flow to the brain results in cell death. Most stroke treatments have targeted acute and subacute stroke, with therapies administered in the first 48 h after onset, as neural tissue is almost completely lost when an infarct transitions from the acute to the chronic stroke phase. For acute stroke, thrombolytic and mechanical thrombolysis are known to improve stroke outcomes. For sub-acute stroke, bone marrow mononuclear cells (BM-MNC) transplantation improves cerebral circulation and stroke outcomes in s murine model (1, 2). Furthermore, clinical trials have reported promising results (3, 4). To date, there is no established treatment for chronic stroke, except rehabilitation. However, rehabilitation alone cannot easily achieve a dramatic recovery in function, which indicates the need for the development of new treatments.

In our previous study, we showed that the transfer of low molecular weight substances, including glucose, from transplanted BM-MNC to cerebral endothelial cells is the prominent pathway in the activation of endothelial cells by BM-MNC transplantation after cerebral ischemia (5). In addition, the transplant of BM-MNC restored the neurological function by activating transcription of major energy supply and consumption-related genes in the brain (6). Therefore, we hypothesized that BM-MNC might have a therapeutic effect in the case of the chronic phase of stroke. In this study, we investigated the effect of BM-MNC transplantation with training, which improves brain plasticity after stroke (7), for chronic stroke.

## Materials and Methods

**Figure 1:**
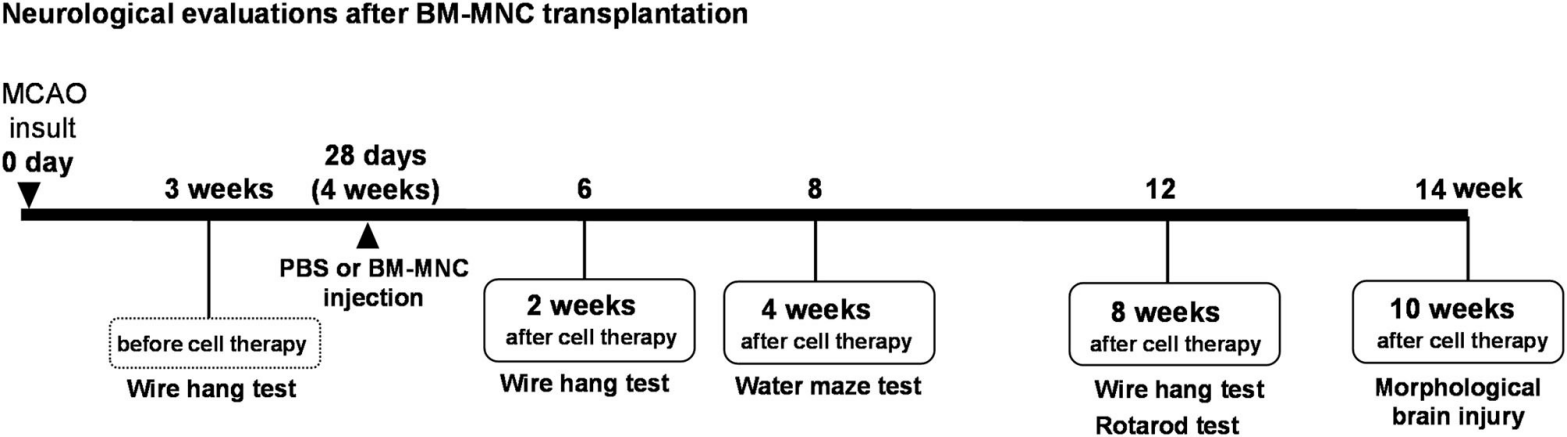
Experimental design. Experimental schedule in this study. MCAO, middle cerebral artery occlusion; BM-MNC, bone marrow mononuclear cells; SCID, severe combined immunodeficiency CB-17/lcr-scid/scidJcl.

This study was approved by the Animal Care and Use Committee of Institute of Biomedical Research and Innovation and complies with the Guide for the Care and Use of Animals published by the Ministry of Education, Culture, Sports, Science, and Technology in Japan. The experimental design is shown in Figure 1.

### Animals

This article adheres to the American Heart Association Journals Implementation of the Transparency and Openness Promotion Guidelines. The data supporting the findings of this study are available from the corresponding author on reasonable request. All animal experiments were approved by the Animal Care and Use Committee of Foundation for Biomedical Research and Innovation and complies with the Guide for the Care and Use of Animals published by the Japanese Ministry of Education, Culture, Sports, Science, and Technology.

### Stroke Model

A murine stroke model with excellent reproducibility was applied in 7-week-old male SCID mice; (CB-17/Icr-scid/scidJcl: Oriental yeast, Tokyo, Japan) as we described previously (2). Briefly, permanent focal cerebral ischemia was induced by permanent ligation and disconnection of the distal portion of the left middle cerebral artery (MCA) using bipolar forceps under Isoflurane inhalation anesthesia (3% for induction and 2% for maintenance). During surgery, rectal temperature was monitored and controlled at 37.0 ± 0.2°C using a feedback-regulated heating pad. Cerebral blood flow (CBF) in the MCA region was also monitored. Mice showing a ≥75% reduction in CBF immediately after MCA occlusion (MCAO) were used for our experiments (success rate, 100%). All mice prepared underwent the surgery successfully. All experiments were performed in a randomized and blinded fashion.

### Preparation of Murine BM-MNC

Mice bone marrow mononuclear cells were prepared as we described previously (8). Briefly, bone marrow of the tibia and femur of 6 weeks old male C57BL/6N mice (Oriental yeast, Tokyo, Japan) was flushed out with phosphate buffered saline (PBS). BM-MNC were isolated by gradient centrifugation using Ficoll-Paque (GE-Healthcare, USA) (9).

### Cell administration

BM-MNC were diluted to the appropriate concentration with PBS and immediately used in our experiments. Four weeks after MCAO procedure, 1 × 10^5^ BM-MNCs in 100 μl PBS or control PBS were injected into the tail vein of SCID mice tail (PBS; *n* = 14, BM-MNCs; *n* = 14) (8).

### Behavioral Tests

The experimental design is shown in Figure 1. To assess motor function, mice were subjected to behavioral testing in the Passive avoidance task, Wire Hang Test, Water Maze Learning, and Rotarod tests before and after MCAO. All behavioral tests were conducted at the optimal time, based on a preliminary study.

#### Passive Avoidance Task

The Passive avoidance task is a fear-aggravated test used to evaluate learning and memory in rodent models of central nervous system disorders. Mice learn to avoid an environment in which an aversive stimulus was previously delivered. we used a device modified from TMS-2 (Meruquest, Toyama, Japan) for this study. The chamber was divided into a light and a dark compartment with a gate between the two. Both compartment sizes were 12.0 cm × 12.0 cm × 13.5 cm. In the conditioning trial, mice were individually placed in the light compartment. Ten seconds later, the door to the dark compartment was opened. When the mouse moved into the dark compartment, the guillotine door was closed, and a scrambled electrical shock (20 mA, 3 s) was delivered 10 s later via the grid floor. Twenty-four hours later, a retention test trial was conducted where no shock was administered. Each mouse was placed in the light compartment and the latency to enter the dark compartment was recorded up to a maximum of 180 s.

#### Wire Hang Test

The wire hang test seeks to evaluates muscular strength or motor function. In this test, each mouse was placed on the wire mesh plate and allowed to accommodate to this environment for 5 s. The wire mesh plate was then gently inverted and secured to the top of a cubic open-topped glass box (25 cm × 25 cm × 25 cm). Latency to fall was measured, with a maximum trial time of 3 min. This trial was repeated five times with an interval of 1 min.

#### Water Maze test

To evaluate learning and memory function, the water maze test was performed. A circular swimming pool (diameter, 100 cm; depth, 40 cm) was placed in a test room and filled with water. A circle plat form (diameter, 8 cm) was submerged 1 cm below the water surface in the center of one quadrant of the pool. Each mouse was subjected to five trials per day for 5 consecutive days, in which they were released into the water with their head facing the outer edge of the pool. The releasing position was any quadrant within which the platform was not hidden. A trial terminated when the mouse reached the platform and remained on it for 3 s. If the platform was not found within 60 s, the mouse was guided to the platform by the experimenter and kept there for 10 s. On the next day of the last training day, all mice were subjected to a probe test trial, in which the platform was removed. Each mouse was released into the south quadrant of the pool and was allowed to swim freely for 60 s. All trials were recorded using video analysis systems (Etho Vision XT5; Noldus, Wageningen, Netherlands).

#### Rotarod Test

Sensorimotor skills were evaluated using the rotarod test. The rotarod drum accelerated from 4 to 40 rpm over 5 min (Muromachi Kikai Co., Ltd., Tokyo, Japan). On the 1st trial (day 1 of 8 weeks), each mouse was placed on the stationary drum. The rotor was started 5 s later. The time until the mouse fell off the rotating drum was recorded. This trial was repeated five times with 1 min intervals. The mean time to fall off the drum over the five trials was calculated. For three days from the first test, training was performed for 5 min every day in all groups. During this training, mice were maintained on the rotating drum (30 rpm) for 5 min. When mice fell, they were returned to the drum for 5 min. This is training aimed at improving the functions of coordination and equilibrium sensation that are impaired by cerebral infarction. The fall latency was re-evaluated at day 5 (day 5 of 8 weeks; 2nd trial).

### Tissue Preparation and Morphology

Ten weeks after cell therapy, mice were sacrificed with pentobarbital i.p. and transcardially perfused with PBS, followed by 4% paraformaldehyde. Subsequently, the whole brain was removed and immersed overnight in the same fixative. Fixed brains were cut coronally into 2-mm slices and the hemispheric volume was estimated by integrating the hemispheric regions. The cerebral hemispheric volume ratio was calculated as the ipsilateral/contralateral hemispheric volume (*n* = 5).

### Data Analysis

Data from the passive avoidance test, wire hang test, water maze test, and rotarod test were assessed using two-way repeated measures analysis of variance (ANOVA), followed by the Bonferroni correction for repeated measure. Differences were considered statistically significant at *p* < 0.05. All results are expressed as the mean ± standard deviation (SD).

## Results

### Passive Avoidance Task: Extending the Transfer Time to the Dark Compartment by BM-MNC Transplantation at 2nd Trial

**Figure 2:**
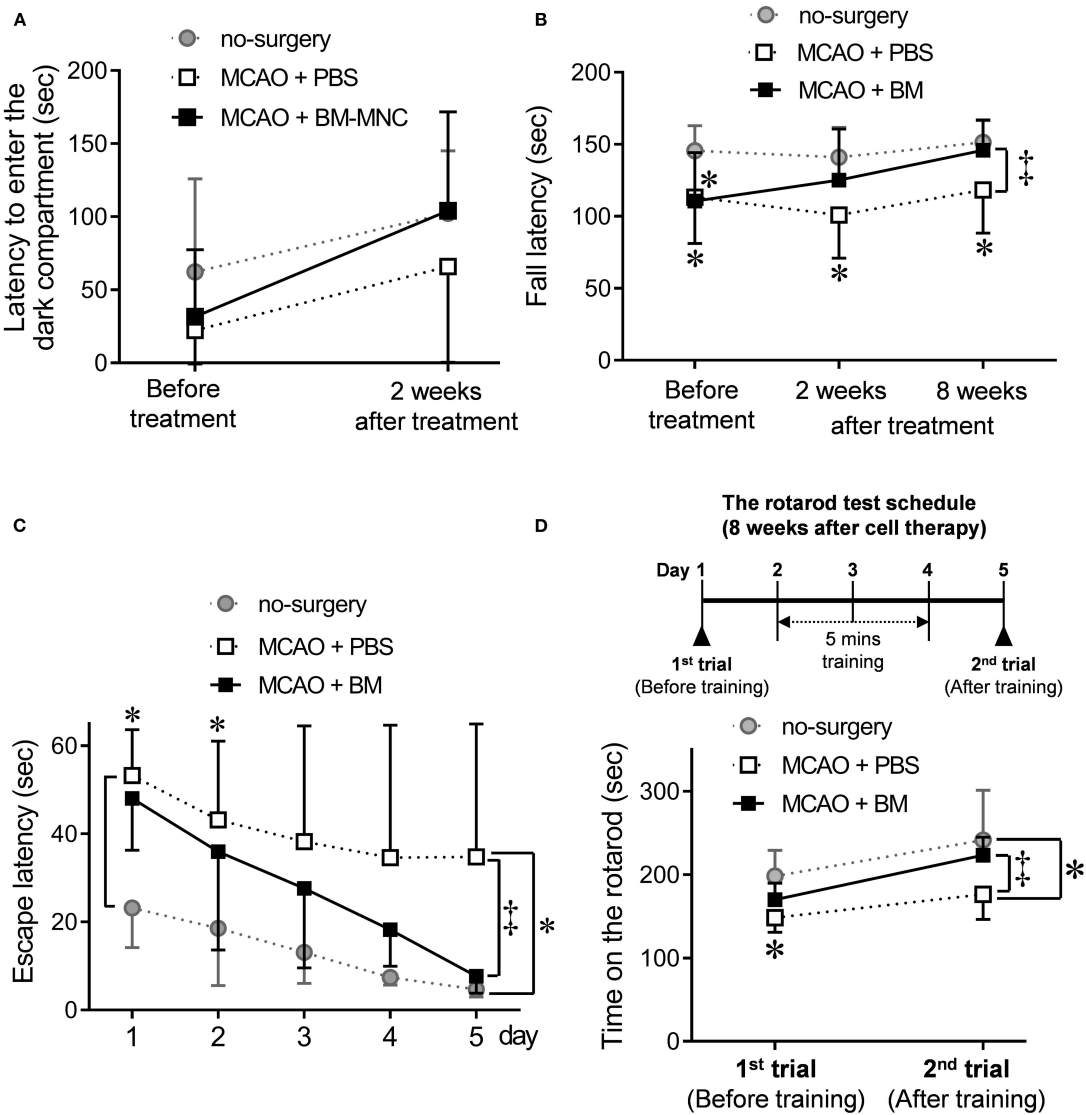
Effect of BM-MNC transplantation at the chronic stroke phase before and after training. **(A)** To assess short-and long-term memory, passive avoidance was used at before the treatment, and 2 weeks after BM-MNC treatment. The latency to enter the dark compartment was measured. The BM-MNC group showed a tendency to a similar latency when compared with the no-surgery group. No significant difference was observed between PBS and BM-MNC-treated mice. **(B)** Neuromuscular function was measured using the wire hang test at before, and 2 and 8 weeks after cell therapy. The latency to fall was measured, and the graph shows the time series variation for this test. No difference was observed between PBS and BM-MNC treated MCAO groups before treatment; however, the latency to fall of BM-MNC treated group at 8 weeks after treatment was similar to the no-surgery mice and significantly longer when compared with the PBS treated mice. **(C)** Water maze test was performed at 4 weeks after the treatment. Time until mice reached a submerged platform was measured for five consecutive days. The mean latency to platform for each day over five sessions is presented. **(D)** Sensorimotor skills were evaluated in the rotarod test. Rotarod performance was defined as the length of time on the rotarod. This rotarod test was performed twice, days 1 and 4 at 8 weeks after treatment. Five minutes of training was conducted daily between the first and second trials. No significant difference was observed between PBS and BM-MNC treated mice; however, 3-days' training significantly improved rotarod performance in the BM-MNC-treated mice; there was a significant difference between the PBS and BM-MNC-treated group. MCAO means permanent middle cerebral artery occlusion model. MCAO + PBS; PBS administration (non-treated) model group, MCAO + BM-MNC; BM-MNC treatment model group. * means no-surgery control vs. MCAO + PBS or MCAO + BM-MNC. ^‡^ means MCAO + PBS vs. MCAO + BM-MNC. ^*‡^*p* < 0.05.

Passive avoidance was performed before and 2 weeks after the cell therapy to evaluate the effect of BM-MNC. At the stage before treatment, two MCAO groups (PBS and BM-MNC treatment) were tendencies to move to dark compartment in a shorter time compared with no-surgery control. There was no difference between two MCAO groups. BM-MNC group showed the tendency to be closer to the no-surgery group by performing cell therapy. However, no significant difference was obtained, since there were large individual differences (Figure 2A).

### Wire Hang Test: Longer Fall Latency at 3rd Trial After BM-MNC Transplantation

We used the wire hang test at before the treatment and 2 and 8 weeks after treatment to evaluate neuromuscular function at the chronic stroke, (Figure 2B). Before the treatment, there was a significantly shorter hanging time in the two MCAO groups (PBS and BM-MNC) when compared with the no-surgery group, and no difference in hanging time between the MCAO groups. There was a tendency toward increased hanging time in the BM-MNC treatment group as the number of tests increased at 2 and 8 weeks. After 8 weeks treatment, there was a significantly increased hanging time in BM-MNC-treated group when compared with the PBS-treated group. These results indicate that the motor function is improved by repeating the test in BM-MNC treated group, although there is no difference in the PBS administration group even if the test is repeated.

### Water Maze Test: Shortening of Escape Latency by BM-MNC Transplantation at 5th Trial

To investigate the spatial learning ability and escape latency, mice performed to water maze test at 4 weeks after cell therapy (Figure 2C). There was no significant difference between PBS- and BM-MNC-treated mice on days 1–4. At day 5th trial, significant shortening of escape latency was observed in mice that received BM-MNC, compared to mice with PBS injection. These results indicated escape reaction and spatial learning ability were significantly improved by BM-MNC transplantation with repeat training.

### Rotarod Test: Elongation of Fall Latency by BM-MNC Transplantation and Repeated Training

Sensorimotor skills and endurance were evaluated at 8 weeks after cell therapy (Figure 2D). There was no significant difference in the fall latency between PBS- and BM-MNC-treated mice on the 1st trial (day 1 of 8 weeks; before training). Following this, mice re-evaluated on day 5 after 3 days' training. There was a significant increase in the latency to fall in BM-MNC-treated mice on the 2nd trial (day 5 of 8 weeks; after training). These results indicated the BM-MNC transplantation significantly enhanced the effect of training, compared with PBS.

### Brain Weight and Morphological Brain Injury

There was no difference in brain weight between PBS and BM-MNC treated chronic MCAO mice. In addition, there was no difference in the ipsilateral and contralateral hemispheric volumes in the PBS-treated group, showed the same tendency in the BM-MNCs (data not shown).

## Discussion

In this study, we have demonstrated the therapeutic effect of BM-MNC on the neurological function in a chronic stroke mouse model. Interestingly, no significant therapeutic effect was observed following BM-MNC administration alone at the chronic stroke phase. However, it became clear that the therapeutic effect became significant by repeating the behavioral tests, in other words, by performing training. Our results indicated that chronic stroke can be a target of cell therapy via BM-MNC transplantation when used in combination with an appropriate training regimen.

BM-MNC transplantation improves outcomes in subacute stroke *via* cerebral circulation and metabolic improvements (2, 3). It has been reported that injured nerve systems are almost completely lost when the infarction transitions from the acute to the chronic phase of stroke. Injured nerve systems are almost completely lost when the infarction transitions from the acute to chronic phase of stroke. Improving the recovery of functions in this chronic stage of tissue loss requires the replacement of multiple elements. It has been argued that stem cells can not accomplish this at the chronic stage. Many studies have measured the effect of BM-MNC in acute or subacute animal models of stroke (1, 10–12); however, to the best of our knowledge, no study has reported a significant therapeutic effect of BM-MNC in a chronic animal stroke model. Previous studies have shown that treatment with BM-MNC at the subacute phase reduces infarction size (13, 14); however, there is no effect on infarct volume with treatment at the chronic stage. This indicates that repair of injured brain volume and improvement of brain function are not equal in the case of chronic brain damage.

Activation of angiogenesis is a key BM-MNC mode of actions (2, 14, 15); both *in vitro* and *in vivo* studies have shown that transplanted cells stimulate angiogenesis and produce many angiogenic factors (16–18). BM-MNC transplantation promotes angiogenesis/neovascularization in the several ischemic diseases, such as limb ischemia and myocardial infarction, even if they are not in the acute phase (19, 20). In addition, we demonstrated that the therapeutic mechanisms of BM-MNC include energy source to injured cells and angiogenesis activation in damaged endothelial cells (6). Therefore, we hypothesized that the therapeutic effect associated with angiogenesis may occur in chronic stroke. Unfortunately, the results of this study showed that angiogenesis alone associated with cell therapy did not lead to any significant improvement in the motor function. However, BM-MNC therapeutic effect became significant by repeating tests even in the chronic phase. In the rodent behavioral evaluation methods, there is no common “training” method. Therefore, in this study, the act of repeating testing/action was defined as training.

Forelimb use is essential to increase the number of pyramidal cell dendritic branches contralateral to lesions in the somatomotor cortex (21). In addition, exercise reduces ischemic brain damage by enhancing neurotrophic expression and regional angiogenesis (22), and enhanced peripheral activity and afferent signals to the ischemic cortex can promote post-ischemic angiogenesis (23). These results indicate that angiogenesis after focal cerebral ischemia can be enhanced by peripheral manipulation. These results indicate that angiogenesis after focal cerebral ischemia can be enhanced by peripheral manipulation. Our study showed that the angiogenesis or motor stimulation alone had no significant effect on the recovery of motor function after stroke. However, we found significant functional improvement following repeated exercise stimulation in combination with angiogenesis. These results indicate that a significant functional improvement can be induced by adding peripheral stimulation to angiogenesis/neovascularization via cell therapy.

In conclusion, our results indicated that the combination of training and cell therapy provides therapeutic benefit in a mouse model of chronic stroke. Further studies on the optimal design and timing of the rehabilitation/training programs at the chronic stroke phase are encouraged. To date, no effective therapeutic option is available at this phase, leading to chronic disabilities. Our findings provide novel therapeutic insights and encourage further studies that assess combination therapies with different targets, such as brain metabolism, neuroplasticity, and reciprocal inhibitory projections, to improve neurological function in chronic stroke.

## Data Availability Statement

All datasets generated for this study are included in the article/supplementary material.

## Ethics Statement

The animal study was reviewed and approved by The Animal Care and Use Committee of Institute of Biomedical Research and Innovation.

## Author Contributions

YOg performed the experiments (behavioral test), analyzed data, and wrote the manusctipt. YOk performed the experiments (animal model). YT, OS, and AK-T supervised the project and revised the manuscript critically for important intellectual content. YOg and AT designed this study. AT prepared this manuscript. All authors gave their approval to the manuscript.

## Conflict of Interest

YOg, YOk, and YT have a patent to patent pending (JP2020/001657). The remaining authors declare that the research was conducted in the absence of any commercial or financial relationships that could be construed as a potential conflict of interest.
